# Identification of several lncRNA-mRNA pairs associated with marbling trait between Nanyang and Angus cattle

**DOI:** 10.1186/s12864-024-10590-x

**Published:** 2024-07-16

**Authors:** Mingyan Shi, Luyao Huang, Shuaitao Meng, Heming Wang, Jinzhou Zhang, Zhiguo Miao, Zhichao Li

**Affiliations:** 1 Life Science College, Luoyang Normal University, Luoyang, Henan, 471934 China; 2 College of Animal Science and Veterinary Medicine, Henan institute of Science and Technology, Xinxiang, 453003 China; 3 College of Animal Science and Technology, Henan Agricultural University, Zhengzhou, 450046 China

**Keywords:** Transcriptome, Marbling, lncRNAs, Target gene, Cattle

## Abstract

**Background:**

The marbling trait of cattle muscles, being a key indicator, played an important role in evaluating beef quality. Two breeds of cattle, namely a high-marbling (Angus) and a low-marbling (Nanyang) one, with their cattle muscles selected as our samples for transcriptome sequencing, were aimed to identify differentially expressed long non-coding RNAs (lncRNAs) and their targets associated with the marbling trait.

**Results:**

Transcriptome sequencing identified 487 and 283 differentially expressed mRNAs and lncRNAs respectively between the high-marbling (Angus) and low-marbling (Nanyang) cattle muscles. Twenty-seven pairs of differentially expressed lncRNAs-mRNAs, including eighteen lncRNAs and eleven target genes, were found to be involved in fat deposition and lipid metabolism. We established a positive correlation between fourteen up-regulated (NONBTAT000849.2, MSTRG.9591.1, NONBTAT031089.1, MSTRG.3720.1, NONBTAT029718.1, NONBTAT004228.2, NONBTAT007494.2, NONBTAT011094.2, NONBTAT015080.2, NONBTAT030943.1, NONBTAT021005.2, NONBTAT021004.2, NONBTAT025985.2, and NONBTAT023845.2) and four down-regulated (NONBTAT000850.2, MSTRG.22188.3, MSTRG.22188.4, and MSTRG.22188.5) lncRNAs and eleven genes related to adiponectin family protein (*ADIPOQ*), cytochrome P450 family (*CYP4V2*), 3-hydroxyacyl-CoA dehydratase family (*HACD4*), kinesin family (*KIF5C*), lipin family (*LPIN2*), perilipin family (*PLIN1*), prostaglandin family (*PTGIS*), solute carrier family (*SLC16A7*, *SLC2213*, and *SLCO4C1*), and containing a transmembrane domain protein family (*VSTM1*).

**Conclusions:**

These candidate genes and lncRNAs can be regarded as being responsible for regulating the marbling trait of cattle. lncRNAs along with the variations in intramuscular fat marbling established a foundation for elucidating the genetic basis of high marbling in cattle.

**Supplementary information:**

The online version contains supplementary material available at 10.1186/s12864-024-10590-x.

## Introduction

The Nanyang cattle are one of the five best cattle breeds in Henan Province, China. In the past, they were known for their large body size and excellent farming abilities, and in today’s commercial economy, they are recognized for their meat performance [1]. The Angus cattle, which originated in Scotland, has tender muscle with a large amount of marbling and is considered one of the most commonly bred cattle. In recent years, consumers have shown greater concern about the quality and acceptability of beef, which are largely determined by the marbling (intramuscular fat content) trait [2]. This trait is also a key indicator for setting the price of beef in the market [3]. Beef with a high intramuscular fat content is favored by consumers due to its superior qualities of tenderness, juiciness, and palatability [4, 5]. From previous studies, some SNPs and related genes involved in the regulation of marbling traits have been identified in pigs and cattle [6–8]. Additionally, a number of genes have been reported to be associated with variations in the marbling phenotype in various species based on transcriptome sequencing, such as intracellular fatty acid binding proteins [9]. Certain biological processes, including fatty acid synthesis, lipid metabolism, and oxidative metabolism, have been found to play crucial roles in the formation of marbling in beef [10, 11]. Furthermore, the expression of specific mRNAs in marble-patterned tissues significantly influences the characteristics of marble patterns and meat quality [12].

lncRNAs, defined as RNA transcripts longer than 200 nucleotides, resemble mRNAs in structure yet cannot encode proteins [13], and they regulate a broad spectrum of biological functions, including cell cycle control, differentiation, apoptosis, chromatin remodeling, and epigenetic regulation [14]. At present, studies have screened out differential lncRNAs related to intramuscular fat deposition in muscle samples of beef cattle and other livestock via transcriptome sequencing, and combined this with functional enrichment pathway analysis to regulate the biological processes of intramuscular fat deposition associated with marbling formation. Research has discovered that pathways related to fat proliferation, fat droplet hypertrophy, and adipocyte differentiation are implicated in the formation of marbling patterns in beef cattle muscle tissue [15]. In Hanwoo beef cattle, three types of samples, namely muscle, intramuscular adipose, and subcutaneous adipose, were taken for RNA sequencing, with the aim of identifying the lncRNAs associated with shear force, body weight, and muscle function [16]. It has been reported that specific metabolic lncRNAs related to fat deposition, like ASMER-1 and ASMER-2, are expressed and enriched in human subcutaneous white adipose tissue and regulated by obesity and insulin resistance, exerting significant roles in adipogenesis and adiponectin secretion [17].

LncRNAs involved in intramuscular fat deposition and marbling have been scarcely explored. Therefore, in this study, we conducted a comparison of the expression pattern based on transcriptome sequenced data between two breeds of cattle. Muscles from a high-marbling (Angus) and a low-marbling (Nanyang) cattle were selected as our samples for transcriptome sequencing, with the aim to identify differentially expressed lncRNAs and their targets associated with the marbling trait.

## Materials and methods

### Ethical statement

All procedures involving animals were approved by the Animal Care and Committee of Henan Institute of Science and Technology. Sample isolation was carried out in accordance with the Animal Care and Use Statute of China.

### Sample tissue collection

Three Nanyang cattle (X15, X25, X35) and three Angus cattle (X13, X23, X51) were selected from the cattle farm of Luoyang Wangzhongwang Cattle Industry Co., Ltd (all cattle are male). According to the marbling grading system of the Chinese Beef Grading Standard (NY/T 676–2003), the three Nanyang cattle samples were classified as the fourth level and set as the low-marbling group, while the three Angus cattle samples were classified as the first level and set as the high-marbling group (Figure S1). The cattle included in the experiment all had the same growth environment and feeding conditions, and all cattle had unrestricted access to water and food. The diet mainly consisted of grass feed, with limited amounts of refined feed. High precision diets, mainly corn, were supplemented three months before slaughter. The cattle were slaughtered at 26 months old (Electrodes were placed on the head and back waist of the cow to form circuits on its body. When power was turned on, the current entered the body of the cow from one electrode and flowed out from the other electrode. High voltage electricity passed through the cow’s body, causing it to become paralyzed and dizzy, followed by bloodletting through the arteries, ultimately resulting in death). The striated muscles of the longissimus dorsi were excised and immediately snap-frozen in liquid nitrogen. The tissues were then homogenized in TRIZOL reagent (Thermo Fisher Scientific, Waltham, MA, USA) using a QIAGEN TissueLyser (QIAGEN, Dusseldorf, NRW, GER) and stored at -80 °C for total RNA extraction.

### RNA extraction and sequencing

After the intramuscular fat was removed from the samples, total RNA was extracted following the manufacturer’s protocol and quantified by a NanoDrop ND-2000 spectrophotometer (Thermo Fisher Scientific, Waltham, MA, USA). The integrity of RNA in each sample was assessed through denaturing agarose gel electrophoresis (Liuyi Biotechnology, Beijing, CHN). RNA sequencing was conducted with 150 bp paired-end sequencing by using a HiSeq 2500 sequencing platform (Illumina, San Diego, CA, USA) at the Shanghai Biotechnology Corporation. The sequencing depth was ≥ 20 Gb.

### Mapping of sequenced reads and lncRNA identification

Raw reads were obtained from the HTSEQ-count (version: 3.0.8) software. Those with a value less than 25 were removed using Seqtk (https://github.com/lh3/seqtk). The high-quality reads were aligned to the cow genome version UMD3.1, which was used as the reference genome downloaded from the Ensembl genome browser (http://grch37.ensembl.org/Cow/Search/Results?q=;site=ensembl;facet_species=Cow), by HISAT2 (version: 2.0.4) [18]. The raw counts were processed and normalized using the DESEq2 software to generate an expression matrix (for mRNA). For lncRNA identification, firstly, we used Stringtie software to assemble transcripts using the alignment results of HISAT2. After removing transcripts with uncertain chain directions, we screened LncRNAs from the remaining set of assembled transcripts. Subsequently, transcripts with lengths below 200 nt and exon counts less than 2 were eliminated. The lncRNAs were screened using four steps: (1) CNCI: coding-non-coding index, which is applied to antisense transcripts and incomplete transcript annotation by using distinguished non-coding and protein-coding transcripts through adjoining a nucleotide triplet independent of known annotations [19]; (2) CPC: coding potential calculator, which is used to calculate protein coding potential by aligning a transcript with a known protein sequence, and a score of < 0 was set as a threshold to screen non-coding RNAs [20]; (3) CPAT: coding potential assessment tool, which distinguishes coding transcripts from non-coding transcripts by calculating Fickett and Hexamer scores based on the length and coverage of the open reading frame [21]; and (4) Pfam: which is used to identify known protein family domains through multiple sequence alignments generated using hidden marker modes, and any transcript without a Pfam hit is a potential lncRNA [22]. Ultimately, the intersection of the outcomes from the four software applications was considered as the identified lncRNA. There are two main approaches to predicting lncRNA targets: cis-acting and trans-acting [69]. To predict cis-acting effects, the co-location threshold was set as 100 kb in both the upstream and downstream regions of lncRNA to identify cis-target genes. Afterwards, the trans-target genes were filtered by examining the association between lncRNA and mRNA expression in the samples (Pearson correlation coefficient, *r* > 0.95).

### Differential expression and functional enrichment

The differential expressed genes among samples were analyzed by edgeR [23]. The expression level was set as log_2_ fold change of FPKM, and the criterion for differentially expressed lncRNAs and protein-coding mRNAs between the two cattle muscles was set as |log_2_ FC fold change| ≥ 1 in the FPKM with *P* ≤ 0.05. Gene Ontology (GO) and Kyoto Encyclopedia of Genes and Genomes (KEGG) pathway analysis were conducted using the Database for Annotation, Visualization and Integrated Discovery (DAVID, http://david.abcc.ncifcrf.gov/). Fisher’s exact test was carried out to select the significant terms with the *P*-value ≤ 0.05. Differential expressed mRNAs, lncRNAs, and their targets were all enriched in the GO and KEGG pathways.

### Co-expression analysis

Cytoscape was employed for complementary energy calculation to identify possible target mRNAs associated with differentially expressed lncRNAs in “trans-target” prediction [24]. The lncRNA-mRNA pairs with an absolute value of *r* > 0.95 and p-value < 0.05 were regarded as significant for co-expression analysis between differentially expressed lncRNAs and their “trans-target” mRNAs when the number of samples was less than 20. Regarding the “cis” regulatory role of lncRNAs, genes at a distance less than 10 kb from lncRNA were considered as possible “cis-targets.” Both types of gene targets, “cis” and “trans,” were subjected to GO analysis and KEGG enrichment.

### Validation of mRNA and lncRNA by qRT-PCR

Quantitative RT-PCR (qRT-PCR) was conducted to verify the expression level of differentially expressed mRNAs and lncRNAs. Briefly, 1 µg of total RNA from the striated muscles of cattle longissimus dorsi was treated with an RNase-free DNase I set, followed by reverse transcription using a ReverAid First Strand cDNA Synthesis kit (Thermo Scientific, USA). Subsequently, qRT-PCR analysis was carried out using a LightCycler 96 (Roch, USA) with SYBR Green master mix (BioRed, USA). The GAPDH gene was employed as a reference gene for normalization and calculated using the 2^−ΔΔ^ Ct method [25]. Genes and primers are listed in supplementary Table S1. Three biological replicates were utilized for qRT-PCR analysis.

## Results

### Overview of transcriptome

A total of 137.11 Gb of clean data were obtained from RNA sequencing (Table 1). The Q20 value of all samples was higher than 95.43%, and the mapping rate ranged from 94.62 to 95.85%. The mapping regions distribution of six samples is shown in Figure S2, and the genome coverage distribution of each sample is shown in Figure S3.

**Table 1:** Summary of transcriptome sequencing and reads mapping

Breed	Group	Sample name	Raw bases (Gb)	Q20 ratio (%)	Clean reads (bp)	Mapping ratio
Nangyang	Low marbling	X15	23.05	95.54%	142,556,920	94.62%
		X25	22.94	95.43%	141,678,788	94.64%
		X35	22.08	95.56%	136,581,478	95.85%
Angus	High marbling	X13	23.52	95.82%	146,538,822	94.63%
		X23	22.33	96.10%	139,564,072	95.66%
		X51	23.25	95.84%	144,437,148	95.84%

**Figure 1:**
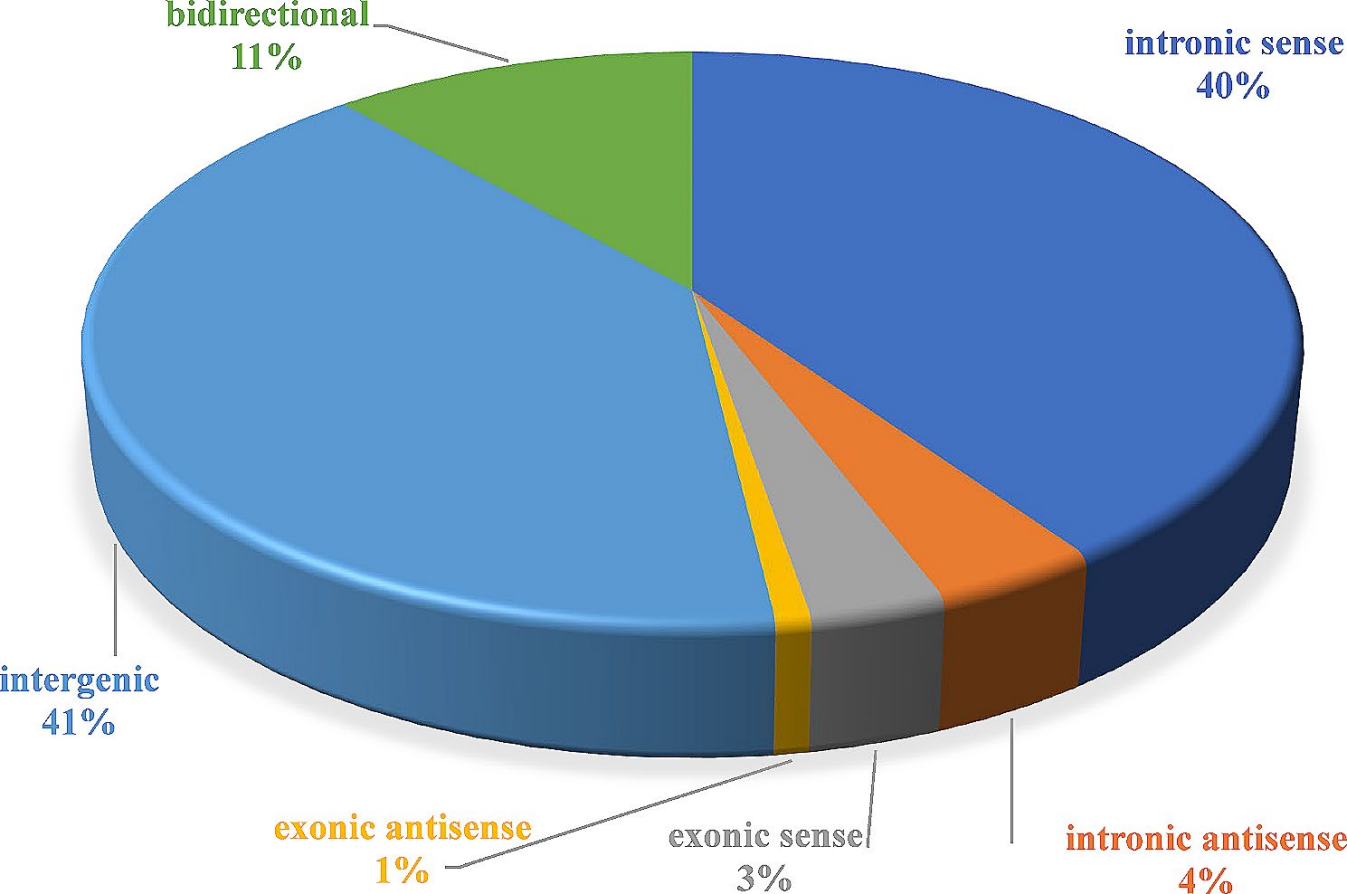
Distribution of identified lncRNAs.

A total of 11,243 lncRNAs transcripts were identified in this study, which could be classified into 4,562 (41%) intergenic, 1,284 (11%) bidirectional, 4,522 (40%) intronic sense, 431 (4%) intronic antisense, 352 (3%) exonic sense, and 92 (1%) exonic antisense (Fig. 1). The transcript lengths of lncRNAs and mRNAs were 342–744 bp and 984-2,549 bp, respectively. The exon number and expression level of lncRNAs and mRNAs were compared in Figure S4.

### Differentially expression mRNAs and lncRNAs

**Figure 2:**
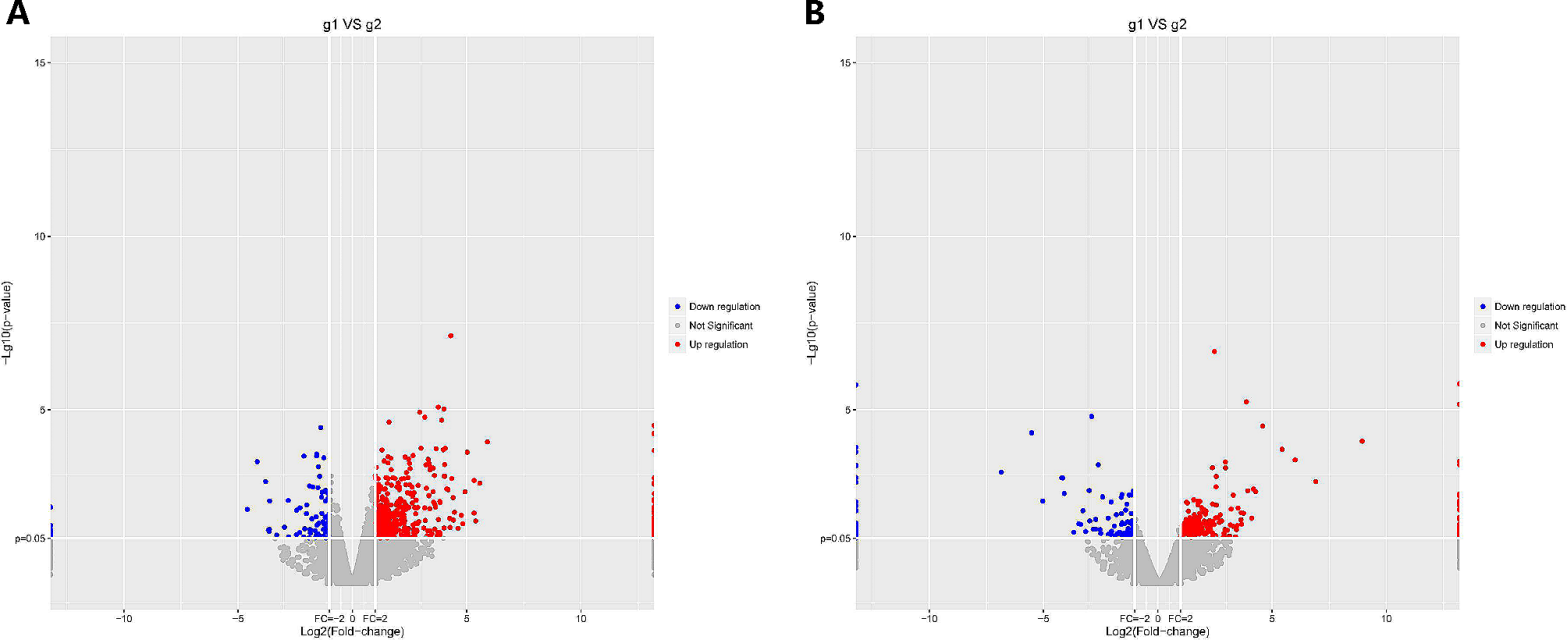
**Volcano plot of differentially expressed a** genes and **b** lncRNAs. The y-axis represents the logarithmic value of Padj, and the x-axis represents the multiple of differences

**Figure 3:**
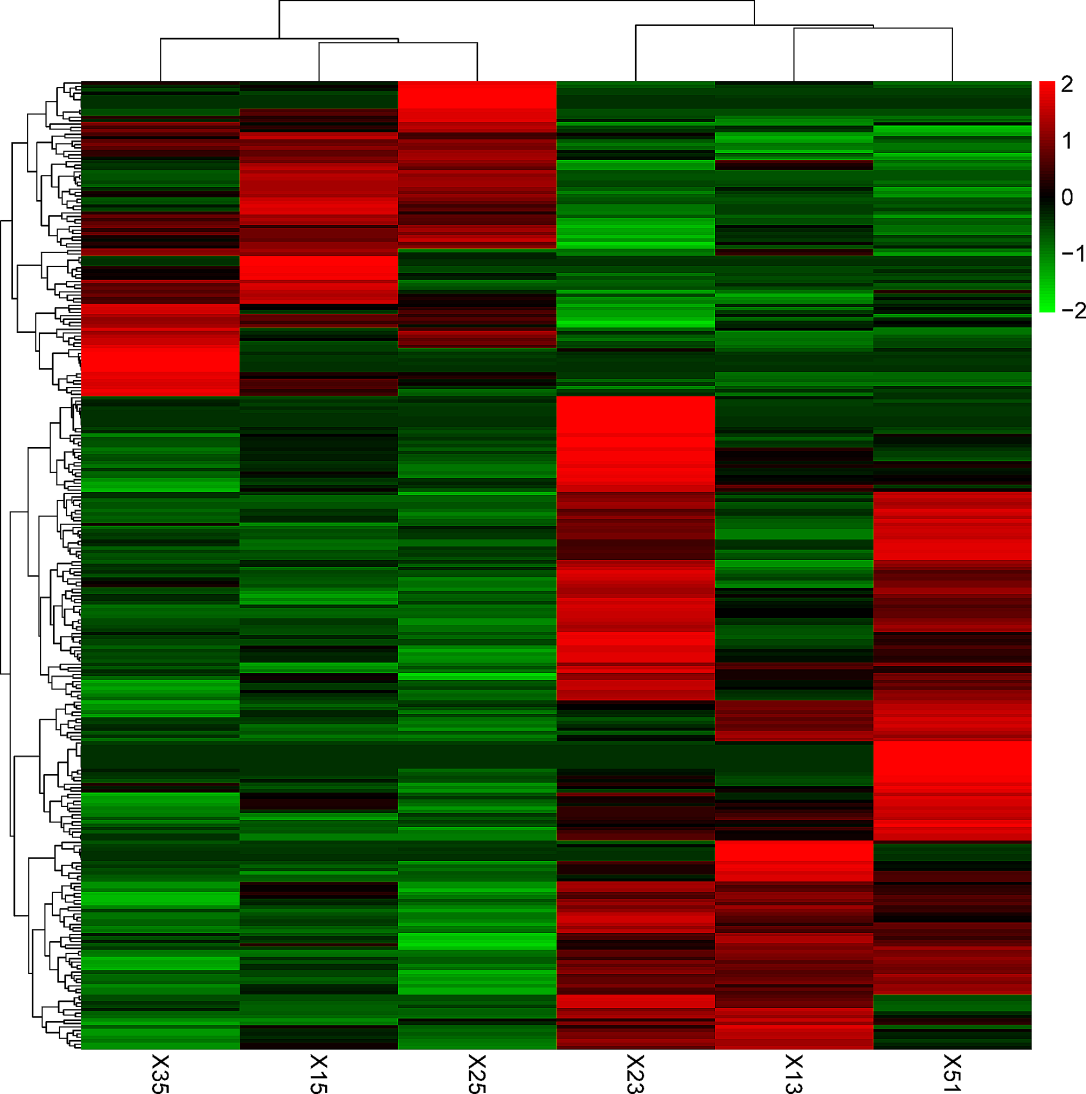
The heatmap of differentially expressed lncRNAs. Each column represents a sample, and each row represents an lncRNA; The expression levels of genes in different samples are represented by different colors, with red indicating high expression and green indicating low expression

A total of 487 differentially expressed mRNAs were detected between two samples. Compared to low-marbling cattle, 411 mRNAs were up-regulated and 76 mRNAs were down-regulated in the high-marbling cattle (Supplementary dataset 1). A total of 283 lncRNAs were identified to be differentially expressed between the high-marbling and low-marbling group, consisting of 191 up-regulated and 92 down-regulated lncRNAs (fold change > 1.0 and *P*-value < 0.05) (Supplement dataset 2 and 3). Among the differentially expressed lncRNAs, the number of intergenic lncRNA, bidirectional lncRNA, intronic sense lncRNA, intronic antisense lncRNA, exonic sense lncRNA, and exonic antisense lncRNA was 125, 46, 81, 11, 14, and 6, respectively. The volcano plot based on differentially expressed mRNAs and lncRNAs showed a certain segmentation both in Nanyang cattle and Angus cattle (Fig. 2a and b). The heatmap of differentially expressed lncRNAs with the average value of FPKM was shown in Fig. 3. The lengths and chromosome distribution of differentially expressed lncRNAs were shown in Figure S5.

### Co-expression analysis of lncRNAs-mRNAs pair to construct relations in pattern of marbling trait development

**Figure 4:**
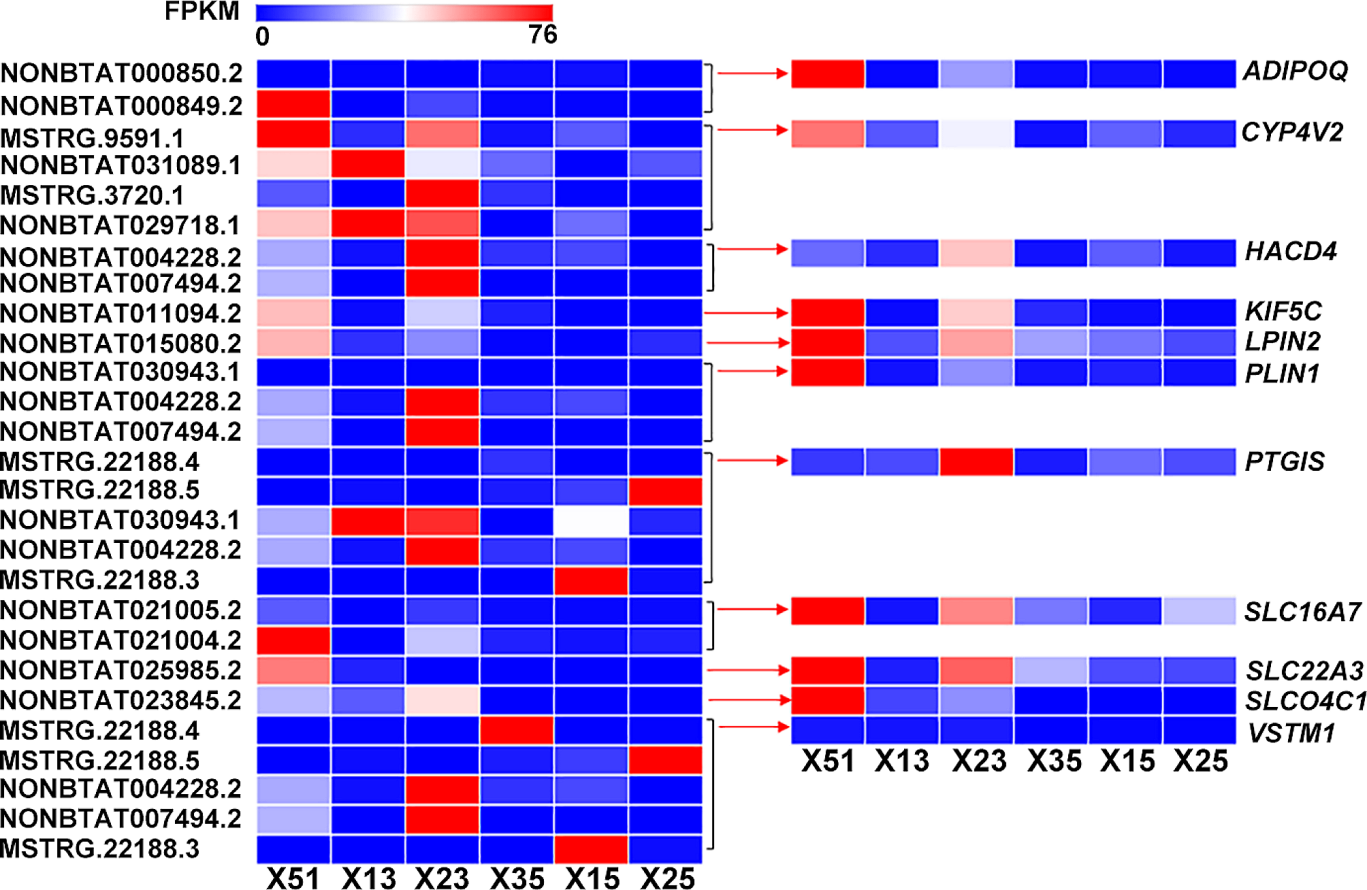
Expression patterns of differentially expressed lncRNA-mRNA pairs related with marbling trait in cattle. Left side: Each column represents a sample, and each row represents an identified lncRNA; The expression levels of genes in different samples are represented by different colors, with red indicating high expression and blue indicating low expression. On the right: Each column represents a sample, and each row represents a target gene for lncRNA; The expression levels of genes in different samples are represented by different colors, with red indicating high expression and blue indicating low expression

The relationship between mRNAs and lncRNAs was established by predicting the same target genes. Among the expression profiles, we found 104 differentially expressed lncRNAs-mRNAs pairs during co-expression analysis, which included 50 differential expression (DE)-lncRNAs that combined with 58 DE-genes (Supplement dataset 4). Of these, 35 differentially expressed lncRNAs-mRNAs pairs were detected to have cis-regulatory activity, and the remaining ones had trans-regulatory activity. The phenomenon of multiple genes regulated by one lncRNA and one gene regulated by multiple lncRNAs were both detected. Eleven genes, including *ADIPOQ* (adiponectin, C1Q and collagen domain containing), *CYP4V2* (cytochrome P450 family 4 subfamily V member 2), *HACD4* (3-hydroxyacyl-CoA dehydratase 4), *KIF5C* (kinesin family member 5 C), *LPIN2* (lipin 2), *PLIN1* (perilipin 1), *PTGIS* (prostaglandin I2 synthase), *VSTM1* (V-set and transmembrane domain containing 1), and three members of the *SLC* (solute carrier) family, were identified and reported to be related to fatty acid metabolic processes and lipid catabolic processes (Table 2). The heatmap of these eleven genes and nineteen regulating lncRNAs is shown in Fig. 4. These target genes mentioned above were all up-regulated under the control of lncRNAs in high-marbling cattle. These lncRNA-mRNAs pairs could be considered as important candidates for further analysis.

**Table 2:** Different expressed lncRNA-mRNA pairs related with lipid metabolism

LncRNA ID	Regulated	Symbol	Regulated	Regulation mode
NONBTAT000849.2	up	ADIPOQ (adiponectin, C1Q and collagen domain containing)	up	cis
NONBTAT000850.2	down			cis
NONBTAT029718.1	up	CYP4V2 (cytochrome P450 family 4 subfamily V member 2)	up	trans
MSTRG.3720.1	up			trans
NONBTAT031089.1	up			trans
MSTRG.9591.1	up			trans
NONBTAT007494.2	up	HACD4 (3-hydroxyacyl-CoA dehydratase 4)	up	trans
NONBTAT004228.2	up			trans
NONBTAT011094.2	up	KIF5C (kinesin family member 5 C)	up	cis
NONBTAT015080.2	up	LPIN2 (lipin 2)	up	cis
NONBTAT030943.1	up	PLIN1 (perilipin 1)	up	trans
NONBTAT004228.2				
NONBTAT007494.2				
NONBTAT004228.2	up	PTGIS (prostaglandin I2)	up	trans
NONBTAT030943.1	up			trans
MSTRG.22188.3	down			trans
MSTRG.22188.5	down			trans
MSTRG.22188.4	down			trans
NONBTAT021005.2	up	SLC16A7 (solute carrier family 16 member 7)	up	cis
NONBTAT021004.2				
NONBTAT025985.2	up	SLC22A3 (solute carrier family 22 member 3)	up	cis
NONBTAT023845.2	up	SLCO4C1 (solute carrier organic anion transporter family member 4C1	up	cis
MSTRG.22188.4	down	VSTM1 (V-set and transmembrane domain containing 1)	up	trans
MSTRG.22188.5	down			
NONBTAT004228.2	up			
NONBTAT007494.2	up			
MSTRG.22188.3	down			

**Figure 5:**
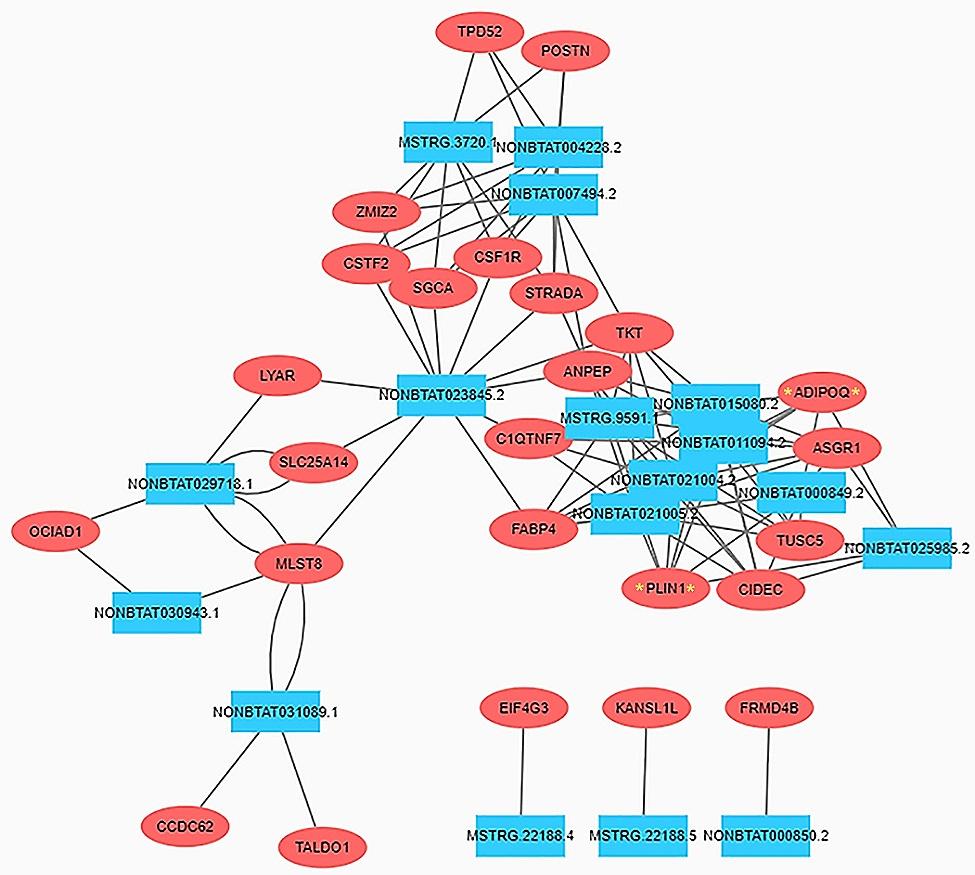
Co-expression analysis for interaction between differentially expressed genes and lncRNAs. Red oval boxes represent genes, while lncRNAs in blue square boxes

All lncRNA-mRNA interactions were described in a network to find their interaction in the biological system through Cytoscape. All lncRNAs that targeted the above eleven genes had a positive synergistic effect on their target genes, but only two genes, *ADIPOQ* and *PLIN1*, related to fatty acid metabolic processes, were detected in this network (Fig. 5).

### Functional annotation of significant differentially expressed genes and target genes of lncRNA

**Figure 6:**
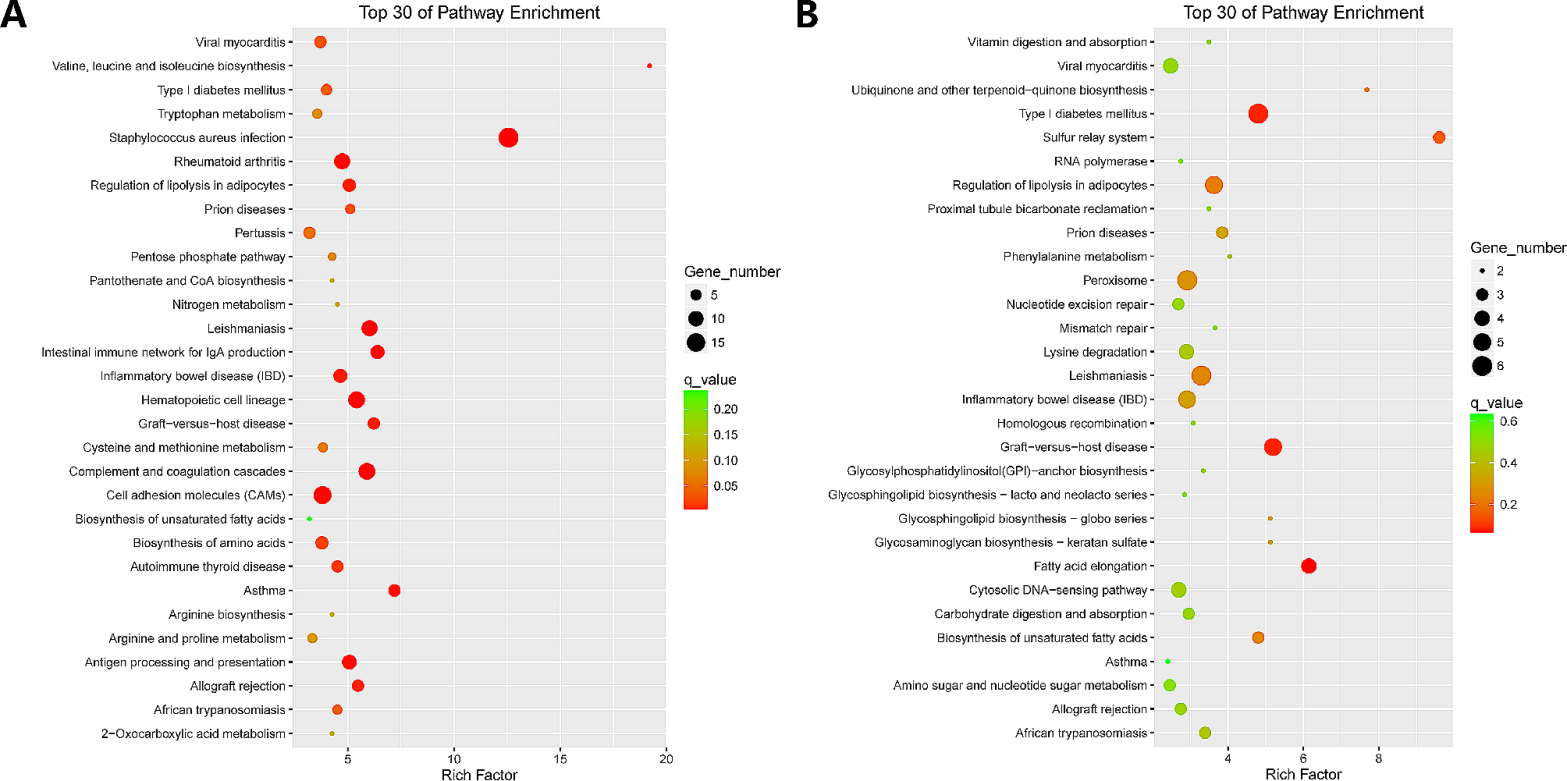
**The differentially expressed of a** genes and **b** lncRNA target genes in top 30 KEGG pathway. The size of the dots represents the number of genes enriched into a specific GO term. The larger the dot, the more genes enriched in the GO term, indicating that the GO term may play a more important role in the studied biological process. The depth of color or the size of - log10 (P-value) is used to indicate the significance of GO terms. The darker the color or the larger the - log10 (P value), the more significant the enrichment of the GO term, indicating that the genes in the GO term are more likely to participate in specific biological processes together compared to the background gene set

GO enrichment analysis of the differentially expressed mRNAs indicated that the terms of positive regulation of inflammatory response, response to interleukin-1, and monocyte chemotaxis in biological processes were mainly enriched (Figure S6a). The differentially expressed mRNAs in the KEGG pathway were largely enriched in the pathways of complement and coagulation cascades, Staphylococcus aureus infection, hematopoietic cell lineage, cell adhesion molecules, cytokine-cytokine receptor interaction, and regulation of lipolysis in adipocytes (Fig. 6a).

GO enrichment analysis of the target genes’ functions of these lncRNAs involved biological processes, including fatty acid biosynthetic process, fatty acid oxidation, lipid oxidation, and tRNA process, were mainly enriched (Figure S6b). The differentially expressed target genes of differentially expressed lncRNAs in the KEGG pathway were largely enriched in the pathways of type I diabetes mellitus, graft-versus-host disease, and fatty acid elongation (Fig. 6b).

### Expression validation of mRNAs and lncRNAs

**Figure 7:**
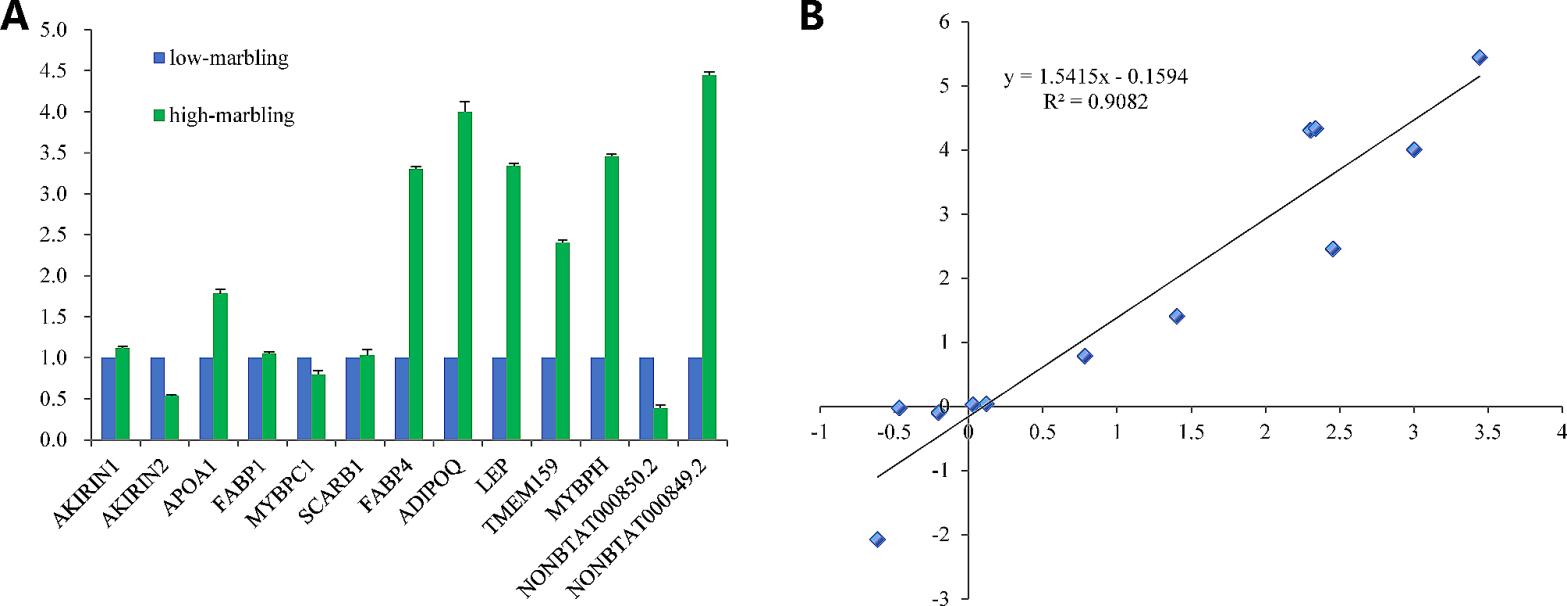
**a** qRT-PCR validation of 13 selected genes between low and high marbling cattle. **b** Correlation analysis of 13 selected genes based on qRT-PCR and RNA-seq data; Pearson’s correlation coefficient R^2^ = 0.9082

To confirm the expression profiles obtained by transcriptome sequencing, eleven differentially expressed mRNAs (*AKIKIN1*, *AKIKIN2*, *APOA1*, *FABP1*, *MYBPC1*, *SCARB1*, *FABP4*, *ADIPOQ*, *LEP*, *TMEM159*, and *MYBPH*) and two differentially expressed lncRNAs (NONBTAT000850.2 and NONBTAT000849.2) that target the ADIPOQ gene were selected for expression validation using qRT-PCR analysis. As expected, the expression pattern of these selected genes revealed by qRT-PCR was consistent with transcriptome sequencing Fig. 7a. The expression trends of all genes from qRT-PCR and RNA-seq analyses were largely consistent (Pearson’s correlation coefficient, R^2^ = 0.9082) (Fig. 7b). This result indicated that our transcriptome sequencing data is reliable.

## Discussion

In the current study, we undertook a comprehensive analysis of differentially expressed lncRNAs through comparing the transcriptome profiles of the high-marbling and low-marbling striated muscle tissues in cattle. A total of 283 differentially expressed lncRNAs and 488 mRNAs were identified, and their general characteristics as well as functional annotations were summarized. Our study provides a comprehensive perception of the molecular mechanisms of lncRNAs associated with meat marbling in cattle.

To our knowledge, several analogous studies on marbling phenotypes have been conducted, and a number of marbling-related genes have been previously identified. In the transcriptome profiling study carried out by Seung-Hwan Lee et al., 21 differentially expressed genes were identified in Korean cattle muscles with diverse marbling phenotypes [26]. In comparison with our present study, it was found that the expression of the common gene *ADAMTS4* was also up-regulated in marbling tissues. Additionally, two common genes, *FABP4* in three breeds of cattle [11] and *CA14* in pigs [2] were found on the list of differentially expressed genes between muscles with diverse marbling phenotypes. *FABP4* was *also* reported to be related to intramuscular fat content in Angus cattle [5]. However, these genes were not detected in the lncRNA-mRNA pairs. Nevertheless, *FABP4* (ENSBTAG00000037526, log_2_FoldChange: 4.30) was detected to be up-regulated in high-marbling cattle only in differentially expressed genes. This result further confirmed the remarkable genetic performance of *FABP4* genes on intramuscular fat deposition, which was also considerable in our experimental cattle.

GO and KEGG pathway analyses were conducted to predict the potential functions of differentially expressed lncRNAs in the longissimus dorsi tissues. The outcomes demonstrated that numerous differentially expressed lncRNAs were annotated to biological functions, such as fatty acid metabolic processes, lipid catabolic processes, and immune-related genes, which are intimately implicated in intramuscular fat deposition [27, 28]. The principal process of IMF deposition and degradation metabolism in beef cattle is the dynamic equilibrium of the transfer, synthesis, and degradation of triglycerides (TG). The synthesis of TG is a crucial factor in IMF deposition, and the raw materials for TG synthesis are mainly non-esterified fatty acids (NEFAs) and carbon chains of glycerol polymers [29]. Research has revealed that newly synthesized fatty acids (FAs) predominantly occur in the adipose tissue of beef cattle, rather than in the liver of ruminant mammals. The FAs utilized for TG synthesis originate from de novo synthesis and degraded fatty acids (FAs) in the diet [30]. There are also reports suggesting that feeding beef cattle with a corn-based diet can enhance the IMF cell uptake of glucose, while a grass-based diet promotes subcutaneous fat deposition by forming FAs using acetic acid as a substrate [31]. This indicated that the expressional alterations in these lncRNAs affected intramuscular fat deposition and marbling phenotypes in cattle. Additionally, beef cattle undergo lipolytic metabolism of IMF and other fats under nutrient negative balance and environmental stress circumstances. The mechanism involves TG generating NEFAs and glycerol (fat mobilization pathway) under lipase hydrolysis. Simultaneously, under environmental stress stimulation, the blood circulation concentration of cortisol hormone (a stress marker) increases, which promotes lipolytic metabolism and results in the enrichment of disease-related pathways [32].

The biological functions of lncRNAs typically involve in the regulation of gene expression. Hence, we focused on differentially expressed lncRNAs and their differentially expressed target genes. A total of 27 lncRNA-mRNA pairs were identified to be involved in fatty acid metabolic processes and related to intramuscular fat deposition (Fig. 4). In our study, *ADIPOQ*, as the target gene regulated by NONBTAT000850.2 and NONBTAT000849.2, showed an upward trend of regulation in high marbling cattle. It has been reported that ADIPOQ is a potential gene for analysis due to its function and biological processes regulating lipid synthesis, glucose utilization, and fatty acid oxidation [33]. Some also believe that ADIPOQ may be beneficial for breeding programs in cattle populations. Therefore, when fat-related traits are regarded as important in commercial production, ADIPOQ may be a valuable functional gene for beef quality traits. Previous studies have demonstrated that lncRNA can regulate fat metabolism processes by directly targeting or indirectly acting on transcription factors (i.e. cis and trans) [34, 35]. Through predicting target genes via trans interactions, it has been reported that lncRNAs such as ENSBTAT 000000 77612, ENSBTAT 000000 72841, ENSBTAT 000000 79684, MSTRG. 9509.3, MSTRG. 9509.2, MSTRG. 1867.3, MSTRG. 2227.1, MSTRG. 9911.1 and others targeting genes related to fat deposition, PLIN1, and ADIPOQ [36, 37]. Through high-throughput sequencing and other methods, it was found that ADIPOQ is an important candidate marker gene for fat deposition in the longissimus dorsi of Angus cattle and Nanyang yellow cattle. Similarly, data indicates that ADIPOQ is a highly expressed candidate gene for bovine fat deposition. Another study suggests that the development of intramuscular fat is positively correlated with the expression of ADIPOQ, while it is negatively correlated with the expression of the myogenic gene MYOD [38]. The above research implies that the molecular function of differentially expressed lncRNAs may be closely related to their target gene ADIPOQ, or may be involved in the regulation of multiple phenotypes. It can be inferred that NONBTAT000850.2 and NONBTAT000 849.2 may regulate intramuscular fat deposition in beef cattle by targeting the expression of ADIPOQ. A fatty acid omega-hydroxylase, named *CYP4V2* [39], was confirmed to be the target of four lncRNAs, including NONBTAT029718.1, MSTRG.3720.1, NONBTAT031089.1, and MSTRG.9591.1. Prostaglandins (*PTGIS*) and fatty acids are physiologically important compounds that are hydroxylated by cytochromes P-450 (*CYP* family) [40]. *PTGIS*, as a target gene, was also found to be simultaneously up-regulated by five lncRNAs, including NONBTAT004228.2, NONBTAT030943.1, MSTRG.22188.3, MSTRG.22188.5, and MSTRG.22188.4, and its modulation of expression character is controlled by adipogenesis synthesis in human subcutaneous and omental adipose tissue [41]. *CYP4V2* and *PTGIS* may jointly play a role in enhancing the marbling trait of cattle. *HACD4* is involved in very long-chain fatty acid synthesi*s* [42] and was targeted by NONBTAT007494.2 and NONBTAT004228.2. Members of the lipin protein family have been reported to have a newly discovered enzymatic role in triglyceride and phospholipid biosynthesis as a phosphatidate phosphatase. For instance, lipin-1 influences lipid metabolism, and lipin-2 and lipin-3 perform similar functions in glycerolipid biosynthesis [43].

*LIPN2* was also found to be up-regulated in high-marbling cattle targeted by the lncRNA of NONBTAT015080.2 in our study. There are reports suggesting that PLIN1 has inhibitory effects on fat breakdown at the levels of gene transcription and protein translation, and its phosphorylation plays a crucial role in fat metabolism and storage; after overexpression of PLIN1, the mRNA expression levels of fat deposition-related genes such as PPARγ, DGAT2, FABP4, and LPL were significantly increased [44], while its knockout would affect the ability of transcription factors to regulate lipid metabolism in bovine adipocytes [45]. Kinesin-1, a member of the kinesin family, was recruited to triglyceride-rich lipid droplets in the liver by the GTPase ARF1 [46]. Another kinesin family member 5 C was precisely detected to have up-regulated expression in high-marbling cattle targeted by NONBTAT011094.2. Sztalryd and Brasaemle reported that *Perilipin 1* controls lipolysis in adipocytes [47]. Three lncRNAs, NONBTAT030943.1, NONBTAT004228.2, and NONBTAT007494.2, targeted the up-regulated *PLIN1* in high-marbling cattle. *SLC25A46* plays a role in a mitochondrial/ER pathway that facilitates lipid transfer [48], and it happened that three SLC family genes (*SLC16A7*, *SLC22A3*, *SLCO4C1*) with up-regulated expression were identified and targeted by the lncRNAs of NONBTAT021005.2, NONBTAT021004.2, NONBTAT025985.2, and NONBTAT023845.2. In addition, another solute carrier family member *SLC8A3* was related to the marbling score in Hanwoo cattle [49]. Transmembrane protein 60 (*TMEM60*) was demonstrated to be associated with increasing marbling fat based on applying the gene co-expression network in cattle [3]. In our study, the *VSTM1* gene containing a transmembrane domain showed an up-regulated expression pattern in high-marbling cattle targeted by five lncRNAs. These aforementioned lncRNAs may be involved in fatty acid metabolism in our study and thus be considered as candidates for further analysis of the lncRNA function in regulating marbling phenotypes.

## Conclusions

In brief, our study revealed a number of lncRNAs and their target genes that play a significant role in regulating fat metabolism via various related pathways, and provided a new perspective for investigating the regulatory mechanism of marbling phenotypes formation in cattle.

### Electronic supplementary material

Below is the link to the electronic supplementary material.

Supplementary Material 1

Supplementary Material 2

Supplementary Material 3

Supplementary Material 4

Supplementary Material 5

Supplementary Material 6

Supplementary Material 7

Supplementary Material 8

Supplementary Material 9

Supplementary Material 10

Supplementary Material 11

Supplementary Material 12

## Data Availability

The raw sequencing data in this study can be found in the Sequence Read Archive under project number PRJNA557260.
