# Supplementary figures and images for: Identification of several lncRNA-mRNA pairs associated with marbling trait between Nanyang and Angus cattle
Source: BMC Genomics. 2024 Jul 16;25:696. doi: 10.1186/s12864-024-10590-x (PMC11250971; doi:10.1186/s12864-024-10590-x)

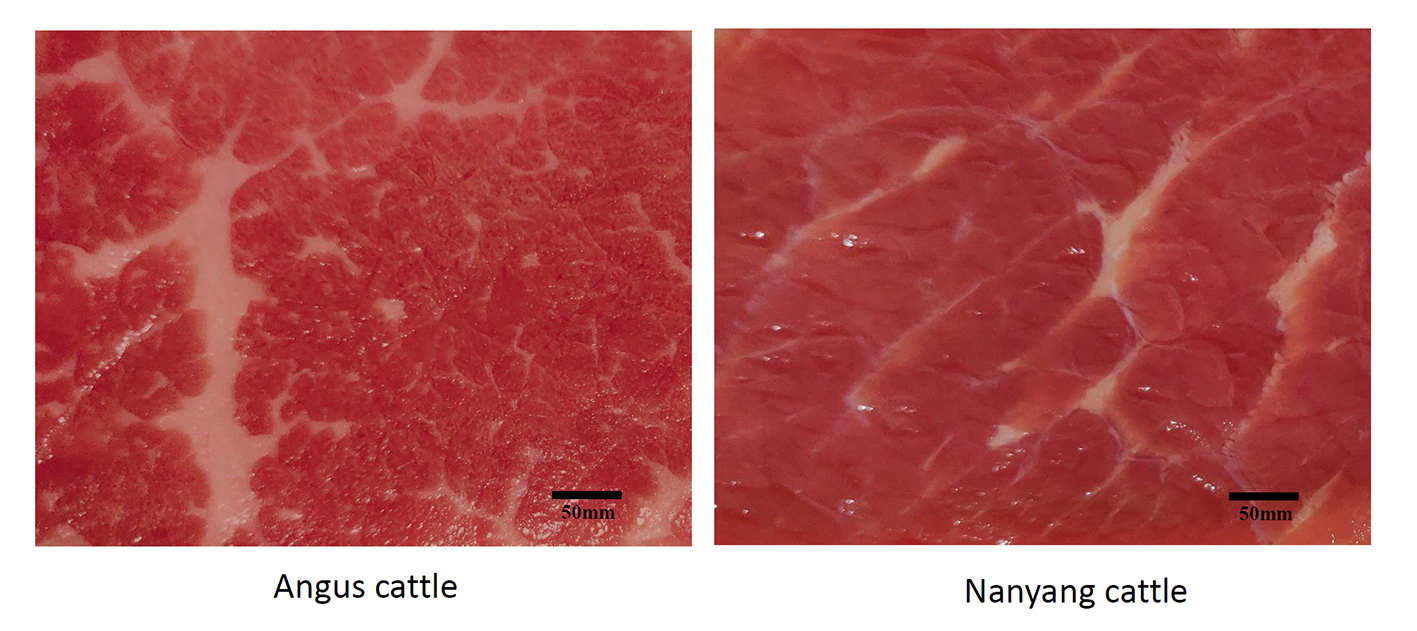

Supplement: Supplementary file 1 — Supplementary Material 1 [file 12864_2024_10590_MOESM1_ESM.tif]

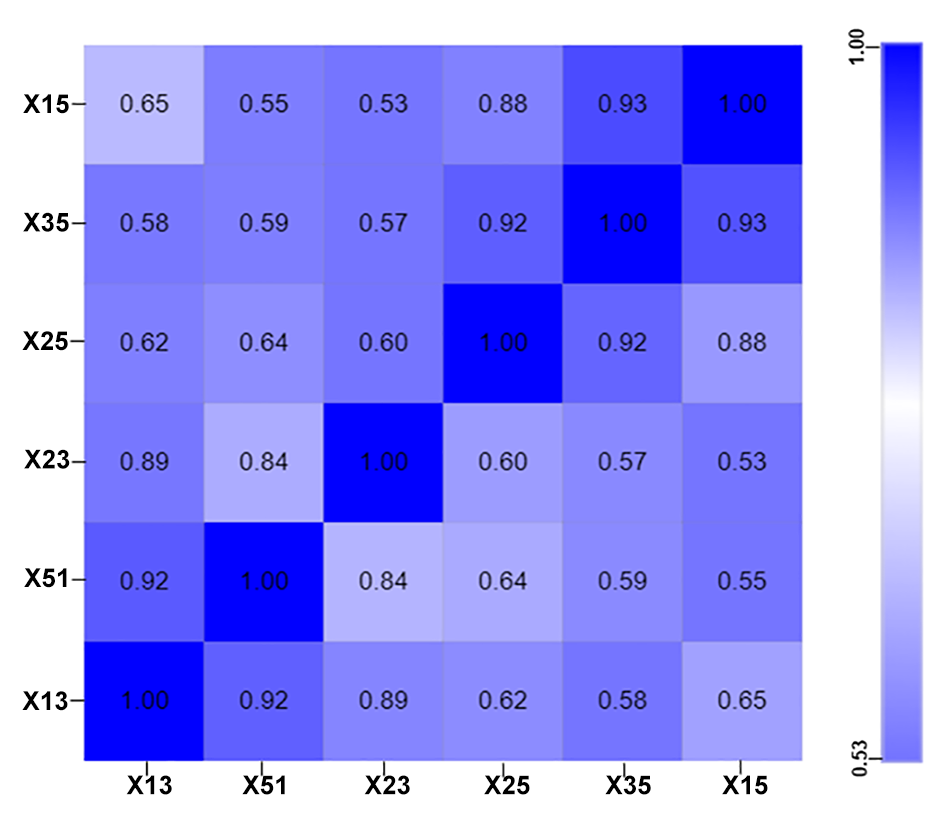

Supplement: Supplementary file 2 — Supplementary Material 2 [file 12864_2024_10590_MOESM2_ESM.tif]

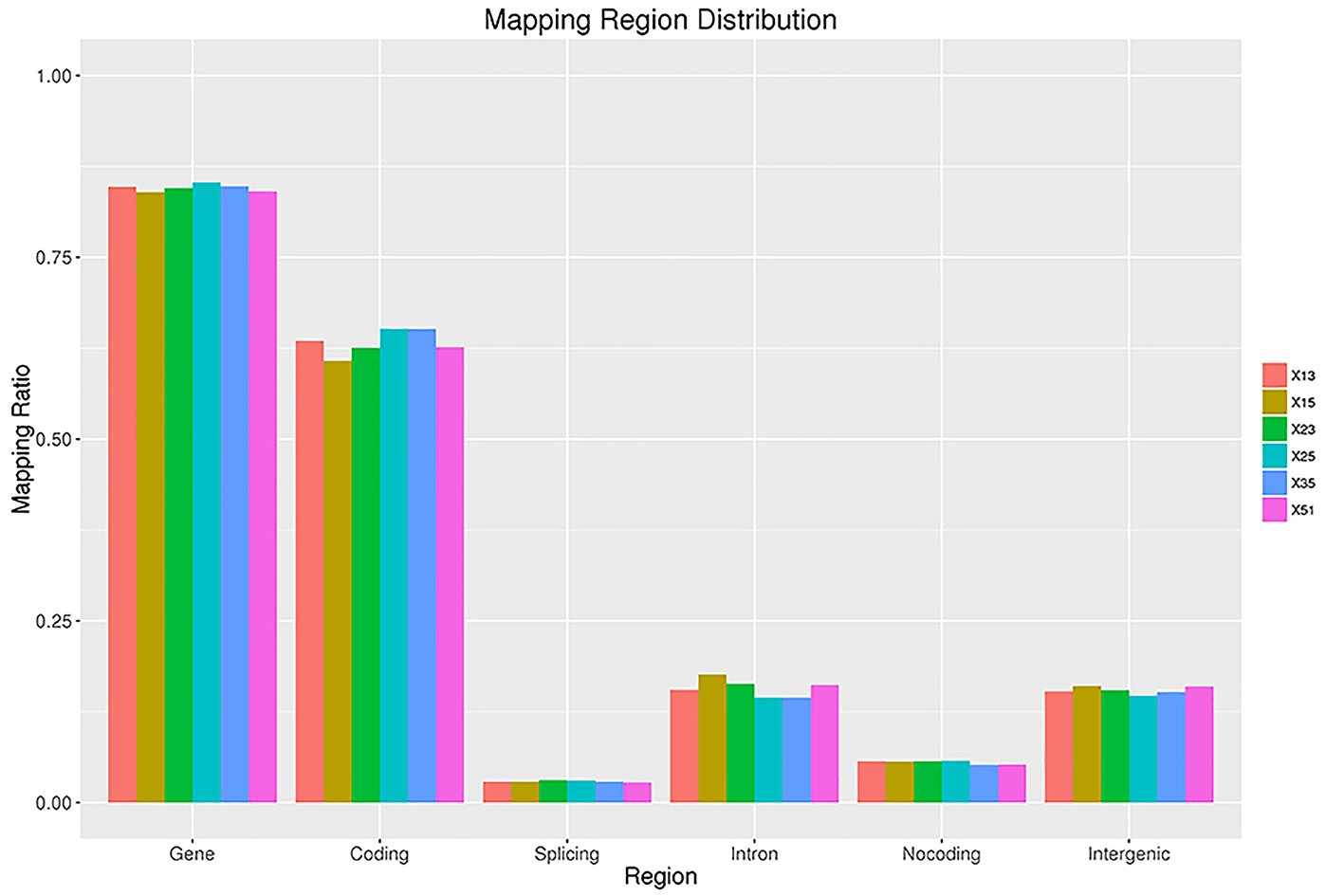

Supplement: Supplementary file 3 — Supplementary Material 3 [file 12864_2024_10590_MOESM3_ESM.tif]

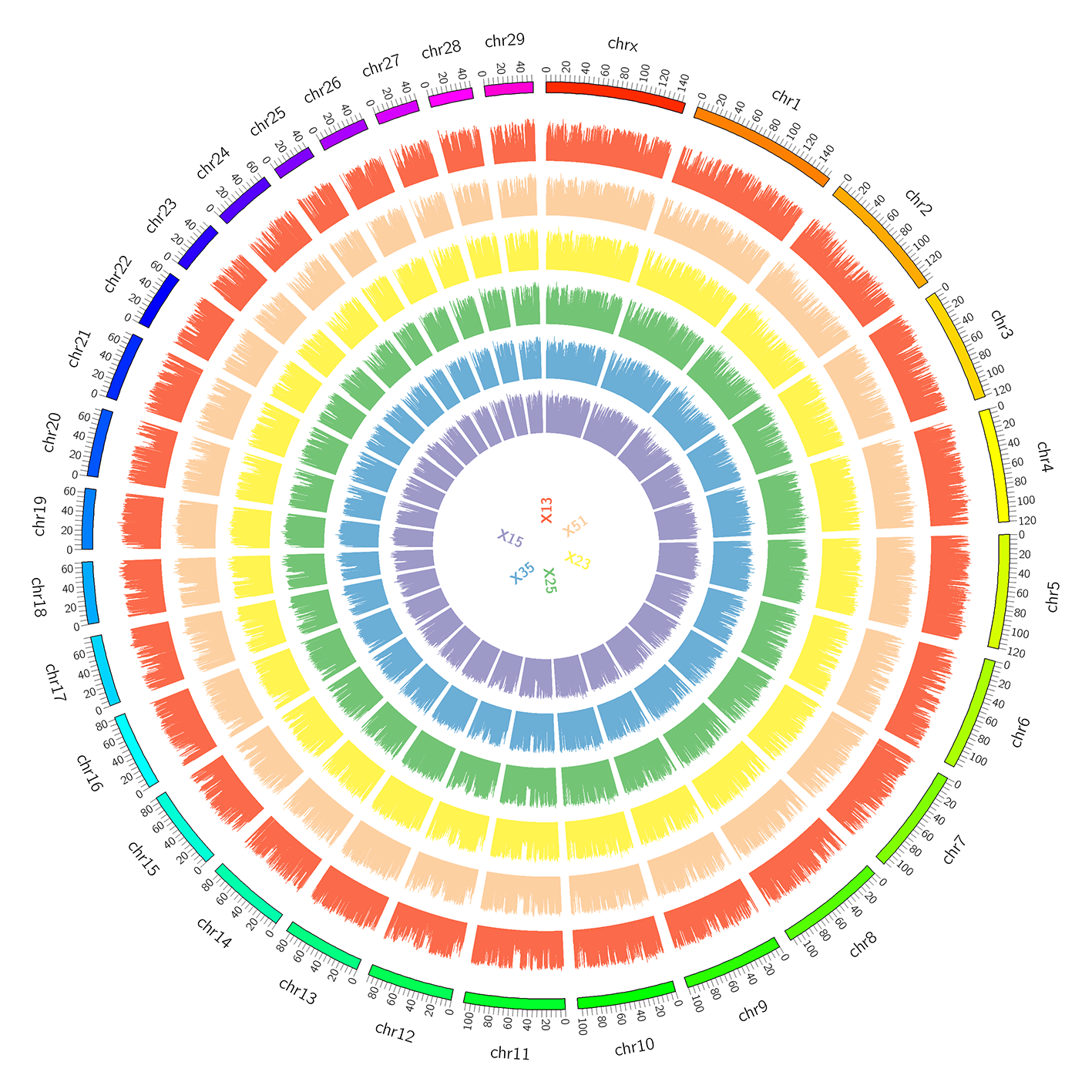

Supplement: Supplementary file 4 — Supplementary Material 4 [file 12864_2024_10590_MOESM4_ESM.tif]

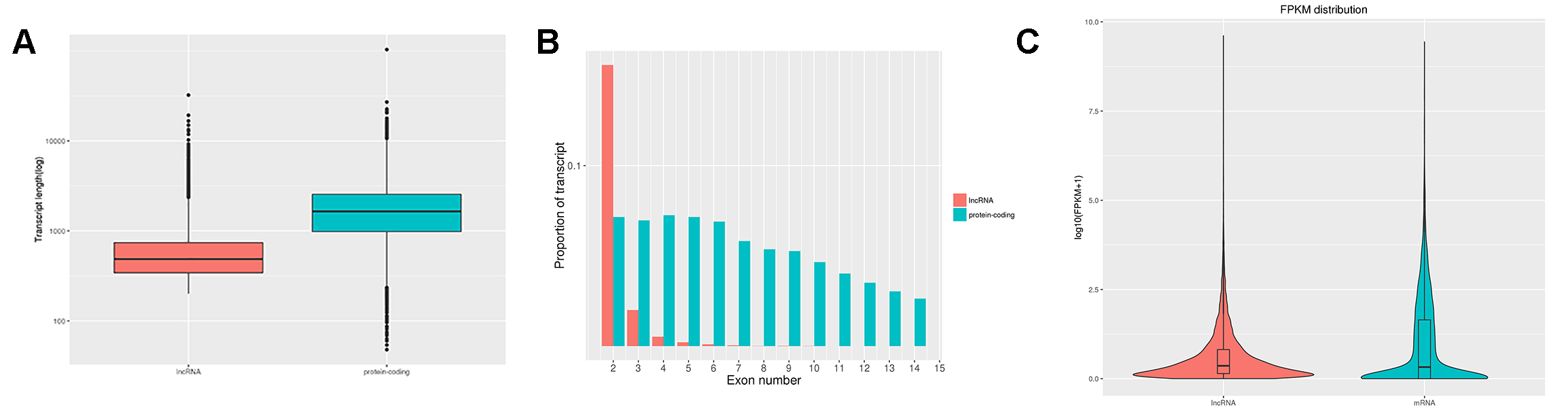

Supplement: Supplementary file 5 — Supplementary Material 5 [file 12864_2024_10590_MOESM5_ESM.tif]

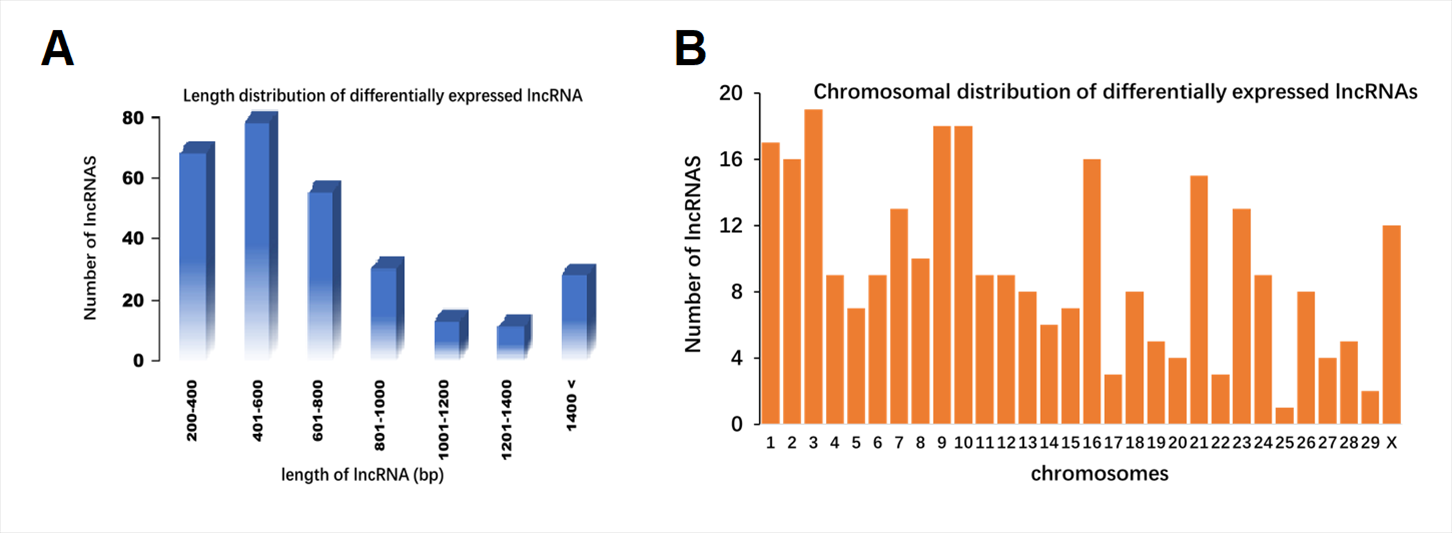

Supplement: Supplementary file 6 — Supplementary Material 6 [file 12864_2024_10590_MOESM6_ESM.tif]

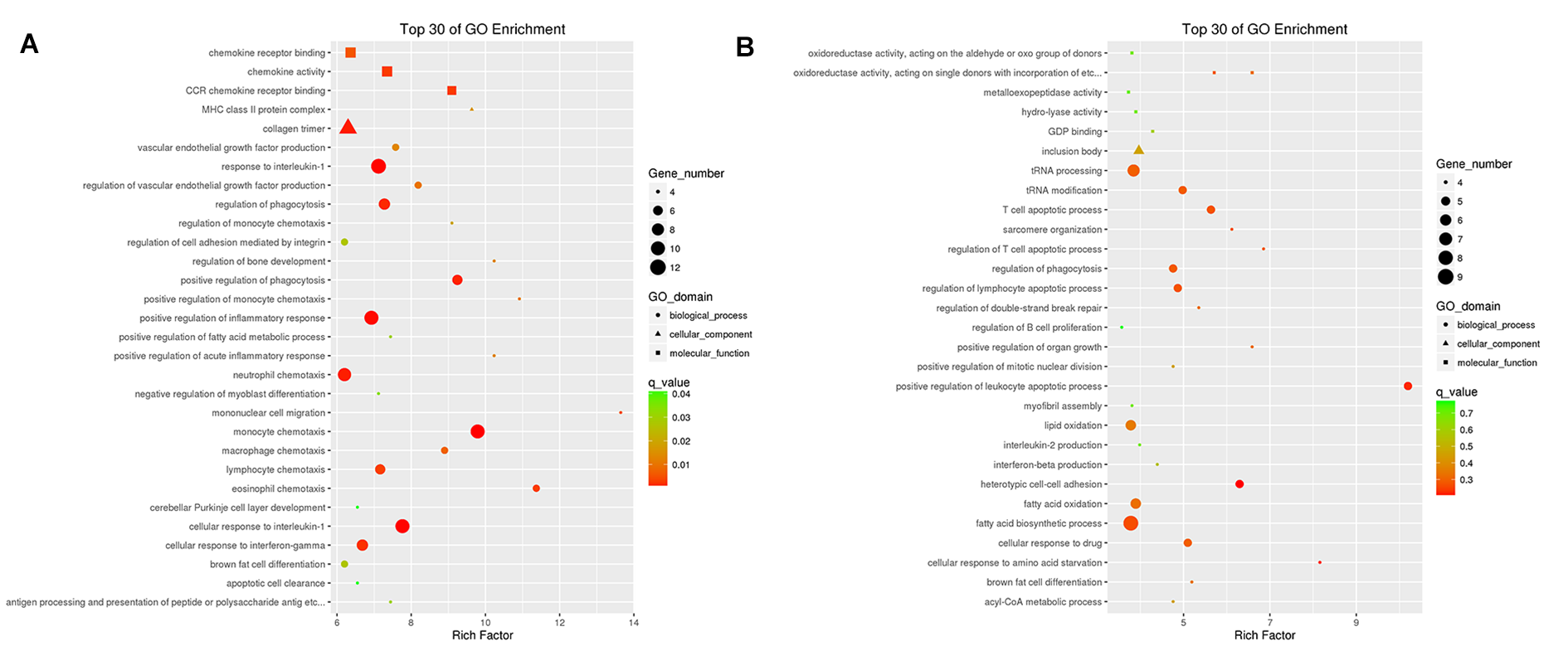

Supplement: Supplementary file 7 — Supplementary Material 7 [file 12864_2024_10590_MOESM7_ESM.tif]
