# Supplementary material for: Identification of several lncRNA-mRNA pairs associated with marbling trait between Nanyang and Angus cattle
Source: BMC Genomics. 2024 Jul 16;25:696. doi: 10.1186/s12864-024-10590-x (PMC11250971; doi:10.1186/s12864-024-10590-x)
Supplement: Supplementary file 12 — Supplementary Material 12 [file 12864_2024_10590_MOESM12_ESM.docx]

Table S1 The primers of the gene and lncRNA for q RT-PCR validtion

| Gene_name | Primer F | Primer R | Amplification length |
| --- | --- | --- | --- |
| AKIRIN1 | GTTCTTAATCAGAGTGAAGCT | AAGATTCATATTGTTCTGCTA | 226 |
| AKIRIN2 | AAGACATTTAGAAACTAGTTTTC | TTCAATATTTCTTCATATTCTT | 231 |
| APOA1 | TCAACCTGAAACTCCTGGACAA | TGTCCTGCAGCTCCTGCACCTT | 294 |
| FABP1 | AGTACCAAGTCCAGACCCAGGAG | TCTCCCCAGTCATGAACTCCAT | 216 |
| MYBPC1 | GTACCTGGAAACTTGTCTCCAGT | TTCCTGTCATCTGGAGTGATTTC | 220 |
| SCARB1 | TAAGTCCCGCGGCCAGGAGAGCG | TGTGAACACGGTGAAGAGGCCAG | 298 |
| FABP4 | TACCTGGAAACTTGTCTCCAGTG | ACTTTCCTGTCATCTGGAGTGAT | 222 |
| ADIPOQ | TTGGTCCTAAGGGTGAGACAG | TCATAATGATTCTGTTGGTTG | 211 |
| LEP | TCGATTCCTGTGGCTTTGGCCCTA | TCCCGGAGGTTCTCCAGGTCATTG | 297 |
| TMEM159 | AGTACTTCAAAGGACTTGCAGGA | CAGCCAGGGAGGTAAGCACCACG | 217 |
| MYBPH | TCGAGAGGGAGCCTTGGACTGGG | AGCCTGAGGCTTGGGATTCCCCT | 289 |
| GAPDH | TGGCAAAGTGGACATCGTCG | TGATGACGAGCTTCCCGTTC |  |
| lncRNA_id |  |  |  |
| NONBTAT000850.2 | ATGCTGCTGCAGGGAGCTCTTC | TCACGGCCTGGTGTGCCATTGT | 152 |
| NONBTAT000849.2 | ATGTGGACCAGGCCTCTGGCT | TTATGGTAGAGAAGGAAGCCT | 148 |
